# Increase in PVC-BSI during the second COVID-19 pandemic year: analysis of catheter and patient characteristics

**DOI:** 10.1186/s13756-024-01476-4

**Published:** 2024-10-08

**Authors:** Eva Pianca, Marie-Céline Zanella, Basilice Minka Obama, Aude Nguyen, Loïc Fortchantre, Marie-Noëlle Chraiti, Stephan Harbarth, Gaud Catho, Aleece MacPhail, Niccolò Buetti

**Affiliations:** 1 Infection Control Program and WHO Collaborating Centre, Faculty of Medicine, Geneva University Hospitals, Geneva, Switzerland; 2 Department of Infectious Diseases, Monash Health, 246 Clayton Road Clayton, Melbourne, 3168 Australia; 3 School of Public Health and Preventive Medicine, Monash University, Melbourne, Australia; 4 Infection Antimicrobials Modeling Evolution (IAME), INSERM, Université Paris-Cité, Paris, U 1137 France

**Keywords:** Catheter-associated bloodstream infection, Peripheral venous catheters, COVID-19, Epidemiology

## Abstract

**Background:**

Increasing nosocomial infections during the COVID-19 pandemic have been reported. However, data describing peripheral venous catheter associated bloodstream infections (PVC-BSI) are limited.

**Aims:**

To describe the epidemiology and risk factors for PVC-BSI during the COVID-19 pandemic.

**Methods:**

We conducted retrospective cohort study of prospectively collected PVC-BSI data in a 2100 bed hospital network in Switzerland. Adult patients with a PVC inserted between January 1, 2020 and December 31, 2021 were included. Risk factors for PVC-BSI were identified through descriptive analysis of patient and catheter characteristics, and univariable marginal Cox models.

**Results:**

206,804 PVCs and 37 PVC-BSI were analysed. Most PVC-BSI were attributed to catheters inserted in the Emergency department (76%) or surgical wards (22%). PVC-BSI increased in 2021 compared to 2020 (hazard ratio 2021 vs. 2020 = 2.73; 95% confidence interval 1.19–6.29), with a numerically higher rate of *Staphylococcus aureus* (1/10, 10%, vs. 5/27, 19%) and polymicrobial infection (0/10, 0% vs. 4/27, 15%). PVC insertions, patient characteristics, and catheter characteristics remained similar across the study period. PVC-BSI risk was associated with admission to the intensive care unit (ICU), and use of wide gauge catheter ( < = 16G).

**Conclusion:**

Increased PVC-BSI during the COVID-19 pandemic was not explained by catheter or patient factors alone, and may result from system-wide changes. PVC-BSI events are primarily attributed to acute care settings, including the emergency department, surgical wards, and the ICU.

**Supplementary Information:**

The online version contains supplementary material available at 10.1186/s13756-024-01476-4.

## Introduction

Peripheral venous catheters (PVC) are the most frequently used invasive devices in hospitalized patients [1]. PVC-associated bloodstream infections (PVC-BSI) are important complications of these devices, leading to major complications including ICU admission and death [2–4].

During the COVID-19 pandemic, increasing rates of nosocomial infections were reported in single-centre studies and surveillance data [5–7]. However, these studies focused on central venous catheters (CVCs) [5, 6]. This reflects more general emphasis in surveillance systems on central line-associated bloodstream infections (CLABSI) relative to PVC-BSI. The relative risk of bloodstream infection is higher for CVCs than PVCs; however, PVCs account for the vast majority of all vascular catheters inserted, and the absolute burden of PVC-BSI may equal that of CLABSI [1]. PVC-BSI have similar mortality risk to CLABSI, and may carry a relatively higher risk of virulent organisms including *Staphylococcus aureus* [8]. An improved understanding of the epidemiology and risk factors of PVC-BSI is therefore essential.

We previously described BSI associated with all catheter types in our 2,100 bed hospital network during the COVID-19 pandemic. We observed an increase in PVC-BSI incidence in 2021 [7], but catheter and patient characteristics were not available in this study, and mechanisms of increased PVC-BSI were not assessed. We therefore developed a large PVC database of over 200,000 catheters to conduct a dedicated cohort study of PVC-BSI between January 2020 and December 2021. We collected additional patient and catheter level data to analyse the mechanisms of increased PVC-BSI. The investigation had two aims: to examine the epidemiology and clinical characteristics of PVC-BSI during this period, and to identify risk factors contributing to the increased incidence.

## Methods

### Study design

We performed a retrospective cohort study from January 1, 2020, to December 31, 2021.

### Setting

Geneva University Hospitals (HUG) is a 10-site, 2100-bed tertiary hospital network in Geneva, Switzerland, receiving approximately 60,000 admissions per year. HUG includes dedicated acute care, rehabilitation, palliative care, geriatric, pediatric, gynecology-obstetric, and psychiatric sites.

Infection prevention measures related to intravenous catheters, and prospective surveillance of catheter related bloodstream infections are described in the supplementary material, and were unchanged across the study period [9].

### Inclusion criteria

All hospitalized adult patients (≥ 18 years old) with at least one PVC inserted during the study period were included. We excluded patients with CVC or long-term catheters including peripherally inserted central catheters, implantable venous access devices and hemodialysis catheters. No midline catheters are inserted at HUG. PVC with a reported dwell time of > 30 days were considered implausible and excluded.

### Outcomes and definitions

The primary outcome was PVC-BSI as defined by two European Centre for Disease Control (ECDC) definitions: Catheter Related Infection for Peripheral Catheters (ECDC-CRI3-PVC), and Hospital Acquired Bloodstream Infection of Peripheral Catheter Origin criteria (ECDC-C-PVC) [10]. Outcome definitions are further described in the supplementary methods.

### Data sources

All hospital onset BSI are investigated by the HUG infection prevention and control (IPC) team as previously described [9]. Hospital onset BSI meeting ECDC criteria were recorded in a hospital database, including ward of acquisition, date of onset, catheter type, surveillance criteria met, and microorganism.

Additional data were extracted from the hospital’s electronic health information system. Catheter data were date of insertion and removal, catheter type, gauge, site, and dwell time. Patient data were date of hospital admission and discharge, sex, and age. Additional data were collected for patients with PVC-BSI, including body mass index, Charlson comorbidity index, immunosuppression, presence of chronic kidney disease and dialysis, SARS-CoV-2, and concomitant infections at other sites.

COVID-19 hospitalisations were extracted from prospectively-collected data from the Swiss hospital sentinel surveillance system [11, 12].

### Statistical analysis

Statistical analysis was performed in three steps. First, we produced a graphically representation of PVC insertions together with PVC-BSI per month across the study period. Second, we investigated yearly PVC-BSI risk per PVC-days (2021 versus 2020) using univariable marginal Cox models on the combined 2020–2021 data set with year of admission as the independent variable of interest. This model takes into account the censored nature of the data and possible intra-cluster dependence using a robust sandwich covariate estimate (PROC PHREG of SAS). Proportionality of hazard risks for catheter type was tested using Martingale residuals. Third, we performed several explanatory analyses. We compared characteristics of PVC inserted in 2021 *versus* 2020, compared characteristics of PVC-BSI in 2021 *versus* 2020, and performed a univariable risk factor analysis for PVC-BSI using marginal Cox models for 2021. Group comparisons were performed using Chi-square tests for equal proportion, students t-test or ANOVA for normally distributed outcomes, and Wilcoxon-Mann-Whitney or Kruskal-Wallis tests otherwise. All statistical analyses were performed using SAS software, version 9.4 (SAS Institute Inc); and Stata, version 18 (StataCorp LLC).

## Results

### Cohort definition

**Fig. 1:**
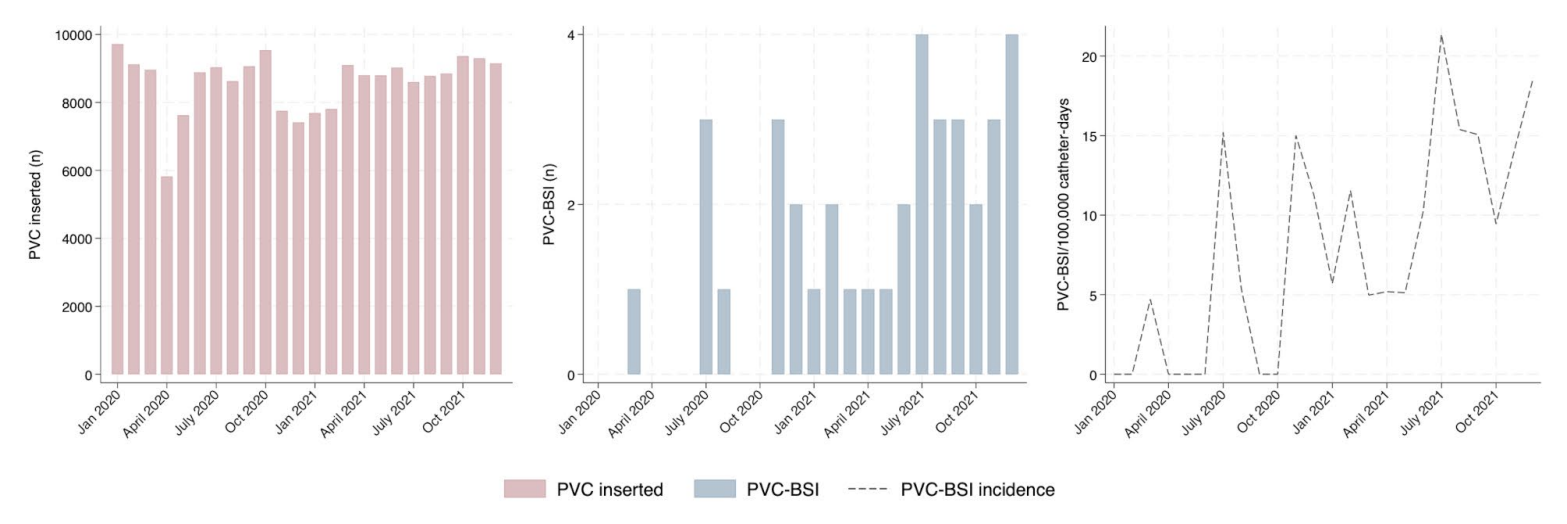
PVC insertions and PVC-BSI per month. PVC Peripheral venous catheter PVC-BSI Peripheral venous catheter-related bloodstream infection

Over the study period, 206,822 PVCs were identified in patients aged ≥ 18 years. Twenty-two PVCs were excluded (reported dwell time > 30 days [*n* = 4]; insertion date prior to January 1, 2020 [*n* = 18]). A total of 206,804 PVCs were included in the final analysis, across 117,337 admissions and 74,711 unique patients (Supplementary e-Fig. 1). Collectively, these represented 467,872 PVC-days over the study period. Monthly frequency of PVC insertions remained stable across the study period, with a few outliers related to the pandemic waves (Fig. 1).

### PVC-BSI rates

A total of 37 PVC-BSI were recorded (Fig. 1). Crude PVC-BSI rates increased from November 2020 onward. Pooled PVC-BSI incidence density was 4.3/100,000 PVC-days in 2020 and 11.5/100,000 PVC-days in 2021. Cumulative incidence of PVC-BSI was 0.01% of all PVCs in 2020 and 0.026% of all PVCs in 2021. In total, 27/37 (73%) of PVC-BSI occurred in 2021.

In univariable Cox models performed on the combined 2020–2021 dataset (population *n* = 206,804 PVCs), the hazard ratio (HR) for PVC-BSI in 2021 vs. 2020 was 2.73 (95% confidence interval [95% CI] 1.19–6.29, *p* = 0.018, proportional hazards assumption was respected).

Increased PVC-BSI rates corresponded with higher overall hospital COVID-19 admissions, which were lowest in May to September 2020, and peaked in October – November 2020. COVID-19 hospitalization numbers at HUG based on Swiss hospital sentinel surveillance data are reported in supplementary e-Fig. 2.

### Changes in PVC and PVC-BSI 2020–2021

Comparisons of PVC inserted in 2020 *versus* 2021 are reported in the supplementary e-Table 1. Overall, PVC inserted in 2021 had similar dwell-time: median 1.7 days (IQR 0.8–3.5 days) in 2020 *versus* median 1.7 days (IQR 0.65–3.5- days) in 2021. Adherence to institutional policy requiring PVC changes within four days of insertion was also unchanged over the study period at 83%. Patients with PVC in 2021 were younger (median 66 *versus* 64 years), and were less likely to be admitted to ICU (3.9% *versus* 1.4%). Distribution of PVC insertion site was similar across the study period. Wide gauge catheters (≤ 16G) were more common in 2020 than 2021 (6.1% vs. 4.6%, supplementary e-Table 1, *p* < 0.01 for all comparisons.)

Characteristics of the subset of PVC associated with PVC-BSI, with comparisons between 2020 *versus* 2021, are reported in supplementary e-Table 2. PVC associated with PVC-BSI in 2020 (*n* = 10) versus 2021 (*n* = 27), showed no significant difference in patient characteristics (age, BMI, or Charlson comorbidity index), nor in catheter characteristics (patient dwell time, time to insertion, insertion site).

Across the study period, PVC-BSI were primarily attributed to catheters inserted in the Emergency department (2020: 8/10 [80%]; 2021: 20/27 [74%]) or surgical wards (2020: 2/10 [20%]; 2021: 6/27 [22%]), with no significant difference between time periods (*p* = 0.81). A substantial proportion of PVC-BSI occurred in PVC with dwell time greater than four days (2020: 5/10 [50%]; 2021: 11/26 [41%]) with no change over the study period (*p* = 0.61).

In 2020, a total of 50% of all PVC-BSI were identified from patients admitted to COVID-19 units with active COVID-19 infection, compared to 18% in 2021, however this did not reach statistical significance (*p* = 0.06).

There were no significant differences in microbiologic etiology between 2020 and 2021. However, we observed a numerically higher rate of infections with *Staphylococcus aureus* (1/10, 10%, vs. 5/27, 19%) and polymicrobial infection (0/10, 0% vs. 4/27, 15%). The proportion of infections caused by other organisms was similar between groups, with coagulase-negative staphylococci most common in both years (38% of all PVC-BSI).

### Risk factor analysis of PVC-BSI

Risk factors for PVC-BSI in 2021 were assessed using a univariable marginal Cox model, reported in Table 1. Risk factors for PVC-BSI included ICU admission (hazard ratio [HR] 33.4, 95% CI 15.5–72.3), and catheter gauge ≤ 16G, (HR 4.80, 95% CI 1.21–19.1). Female sex was associated with reduced risk of PVC-BSI (HR 0.33, 95% CI 0.13–0.8).

**Table 1:** Univariable marginal Cox model for PVC-BSI among all PVC (*n* = 105,254) in 2021

	Without PVC-BSI		PVC-BSI		HR	95% CI	*p*-value
Sex							
Female (%)	54,012	(51.3)	6	(22.2)	0.33	[0.13–0.84]	0.02
Male (%)	51,215	(48.7)	21	(77.8)	.		
Age > 66 years (%)	48,935	(46.5)	15	(55.6)	1.08	[0.50–2.34]	0.85
ICU admission (%)	1,466	(1.4)	9	(33.3)	33.42	[15.45–72.33]	< 0.01
Catheter Size							
<=16G	4,804	(4.6)	5	(20)	4.80	[1.21–19.1]	
18G	41,552	(40)	8	(32)	0.99	[0.21–4.64]	
20G	48,679	(46.9)	10	(40)	0.90	[0.2–4.07]	
>=22G	8,726	(8.4)	2	(8)	Reference		0.01
Time to insertion from admission, median (IQR)	1	[1 ; 4]	2	(1, 5)	1.00	[0.98–1.01]	0.40
Insertion site							
Arm	1,801	(1.7)	1	(3.7)	2.54	[0.27–24.22]	
Cubital fossa	21,925	(20.8)	4	(14.8)	1.26	[0.28–5.68]	
Foream	38,455	(36.5)	9	(33.3)	1.27	[0.34–4.71]	
Hand	23,407	(22.2)	10	(37)	3.30	[0.91-12]	
Other	19,639	(18.7)	3	(11.1)	Reference		0.15

ICU intensive care unit IQR interquartile range PVC peripheral venous catheter PVC-BSI peripheral venous catheter-associated bloodstream infection

Risk factor analysis for PVC-BSI in 2020 was also performed using univariable marginal Cox model. Results were limited by low event numbers. Only ICU admission was associated with increased risk of PVC-BSI (HR 6.72, 95% CI 1.29–34.9, supplementary e-Table 3). In univariable marginal Cox model performed on the combined data (2020–2021) only ICU admission, sex and year (2021 vs. 2020) were significant (supplementary e-Table 4).

## Discussion

We analysed patient and catheter characteristics in a retrospective cohort of more than 200,000 PVCs to identify mechanisms of increased PVC-BSI during the second year of the COVID-19 pandemic. We undertook dedicated prospective surveillance of all hospital-onset BSI and identified an increase in PVC-BSI between 2020 and 2021. In 2020, at the beginning of pandemic conditions in Switzerland, absolute COVID case numbers were lower in our institution; in 2021, PVC-BSI increased in line with rising COVID-19 case numbers. In our analysis of patient and catheter characteristics, we did not identify significant differences that would explain this increase, and only a minority of patients with PVC-BSI were admitted to dedicated COVID-19 units. Taken collectively, this may suggest institutional, systems-level factors to be responsible for increased PVC-BSI during pandemic conditions. Possible explanations include diversion of IPC resources and expertise, redeployment of staff to new envionments, multiple rapid changes in IPC practice, and strain on hospital resources, workload and staffing [13].

Very few previous data describing PVC-BSI are available for comparison. Regarding central lines, increased CLABSI during the COVID-19 pandemic, have been reported in single-centre studies and routine surveillance data [5, 6, 14–16]. These reports include surveillance data from the United States reported to the Centers for Disease Control and Prevention/National Healthcare Safety Network [5, 15], and from the Dutch national surveillance system PREZIES [17]. In addition, a consortium of seven low and middle income countries reporting data to the International Nosocomial Infection Control Consortium (India, Mongolia, Jordan, Lebanon, Palestine, Egypt, and Turkey) reported increased CLABSI rates after the beginning of the COVID-19 pandemic [14]. Dedicated single centre studies in the United States [6] and Europe [16] have also reported a significant increase in CLABSI rates. However, these did not include PVC, and primarily report data from 2020 only. Additionally, most studies include very limited patient- and catheter-related data. These reports do not investigate specific causative mechanisms for the changes in hospital acquired infections during pandemic conditions.

In our institution, only a minority of PVC-BSI were reported from patients admitted to dedicated COVID-19 wards, or with active COVID-19 infection. This is in contrast to one single-centre cohort study in the United States, which reported increased CLABSI risk in patients with COVID-19, but no difference in CLABSI rates in the non-COVID hospital population [6]. However, other multi-centre studies have described increased non-COVID mortality relating to global effects of pandemic conditions, including hospital strain, with non-COVID mortality rising in proportion to COVID-19 admission rates [13, 18].

We identified several patient groups at elevated risk of PVC-BSI. Admission to ICU and wide-gauge catheter were associated with increased risk of infection. In addition, more than 95% of all PVC-BSI occurred in catheters inserted either in the emergency department, or surgical wards. This is consistent with previous findings of increased PVC-BSI risk with wide-gauge catheters [19]. These data suggest that acutely unwell patients, who may have catheters inserted in emergency conditions, are at higher risk and represent a target group for prevention. However, both ICU admission and wide-gauge catheter were more common in 2020 than 2021, and therefore cannot explain the rise in PVC-BSI over this study period.

Patients with prolonged PVC-dwell time were also disproportionately represented in the PVC-BSI group. Routine PVC change every four days was recommended in our institution over the study period. Adherence to this policy was around 83%, but approximately half of all PVC-BSI occurred in patients with PVC dwell time above four days. Adherence to PVC replacement was stable and does not explain the increase in PVC-BSI over the COVID period, but may be a target to reduce PVC-BSI risk [9].

Our study has several limitations. First, this investigation may be under-powered to detect differences in catheter- or patient-related factors that contribute to changing risk. Second, we describe only PVC-BSI in the first two years of the COVID-19 pandemic. We did not compare pre- and post-pandemic conditions. The study period was determined by a previous observation of increased PVC-BSI rates during the alpha and delta waves in 2021 [7] Pre-pandemic analysis was not possible, as prior to 2020 we conducted an intervention investigating routine versus clinically indicated PVC replacement, which would have confounded our observations [9]. Further analyses that include the pre-pandemic and post-pandemic periods (2022 onward) are needed to better understand the impact of pandemic conditions.

Third, due to low incidence of intravascular catheter infections, we fitted only *univariable* Cox models to identify PVC-BSI risk factors. Multivariable analysis was not performed due to low event number. This increases the risk of confounding and precludes analysis of interactions between different risk factors; the results of this analysis should therefore be interpreted cautiously.

Finally, potentially contributory hospital factors such as bed occupancy, staff absences, hospital systems changes and compliance with infection prevention measures, were not assessed. We hypothesise that system-wide changes in the first two years of the COVID-19 pandemic contributed to increasing risk of nosocomial infection. In March 2020, the HUG hospital network was restructured to allow admission of large numbers of COVID-19 patients. This included: transformation of medical units into units dedicated to COVID-19 care; diversion of non-COVID-19 patients to other hospitals in the area; and reassignment of health care workers (HCWs) to different care environments. In this setting, burnout, workforce shortages, multiple rapid changes in procedure and loss of specific expertise due to diversion of staff to new roles may all have contributed to increasing rates of nosocomial infection.

This study has several strengths. Dedicated prospective surveillance of all hospital-onset BSIs was conducted throughout the study period. We applied ECDC surveillance definitions with high specificity, and assessed a range of patient-level clinical data and catheter-related data.

## Conclusions

We observed a significant increase in PVC-BSI in a Swiss hospital network under COVID pandemic conditions in 2021, which was not explained by changes in catheter characteristics or patient demographics. The impact of systemic changes during the COVID-19 pandemic on PVC-BSI warrants further investigation including multivariable and longitudinal analyses. Post-pandemic quality improvement efforts should target PVC care for acutely unwell patients in Emergency Deprartment, ICU and surgical wards.

## Electronic supplementary material

Below is the link to the electronic supplementary material.

Supplementary Material 1

## Data Availability

The datasets generated and/or analysed during the current study are not publicly available due to patient confidentiality. Data may be made available on reasonable request and these can be addressed to the corresponding author.
