# Supplementary material for: Increase in PVC-BSI during the second COVID-19 pandemic year: analysis of catheter and patient characteristics
Source: Antimicrob Resist Infect Control. 2024 Oct 8;13:120. doi: 10.1186/s13756-024-01476-4 (PMC11463163; doi:10.1186/s13756-024-01476-4)
Supplement: Supplementary file 1 — Supplementary Material 1 [file 13756_2024_1476_MOESM1_ESM.docx]

**Increase in PVC-BSI during the second COVID-19 pandemic year: analysis of catheter and patient characteristics**

Eva Pianca*, Marie-Céline Zanella* Basilice Minka Obama, Aude Nguyen, Loïc Fortchantre, Marie-Noëlle Chraiti, Stephan Harbarth, Gaud Catho, Aleece MacPhail*, Niccolò Buetti*

**Supplementary materials and methods**

**Outcome definitions**

The primary outcome was PVC-BSI as defined by European Centre for Disease Control (ECDC) definitions [1]. We included CABSI meeting criteria for ECDC Catheter Related Infection criteria for peripheral catheters (ECDC-CRI3-PVC), and ECDC Hospital Acquired Bloodstream Infection (Peripheral Catheter Origin) criteria (ECDC-C-PVC). The CRI-3 definition requires microbiological confirmation of a line source of infection demonstrated by positive blood culture <48 hours after catheter removal and the same microorganism isolated from a quantitative catheter tip culture of greater than 10^3^ colony forming units (CFU)/mL, or the same microorganism isolated in a culture from pus collected from a catheter site. The C-PVC definition requires isolation of the same organism from peripheral blood and PVC, or improvement of symptoms within 48 hours of removal of a PVC, in the absence of another source.

**Impact of COVID-19 on hospital processes at Geneva University Hospitals (HUG)**

In March 2020, HUG was restructured to allow admission of large numbers of COVID-19 patients. This included: transformation of medical units into units dedicated to COVID-19 care; diversion of non-COVID-19 patients to other hospitals in the area; and reassignment of health care workers (HCWs) to different care environments. COVID-19 hospitalization numbers at HUG is reported in e-Figure 2 and is based on prospectively collected data from the Swiss hospital sentinel surveillance system [2, 3].

Infection prevention measures related to intravenous catheters remained similar across the study period and are described below. Prospective surveillance of BSI also continued unchanged as previously described [4].

**Infection prevention procedures for intravascular catheters**

Institutional recommendations for preventing PVC-associated infections were the following: (i) skin antisepsis before catheter insertion and during dressing changes with alcohol-containing 2% chlorhexidine-gluconate (CHG); (ii) the selection of site of insertion was left to the discretion of the HCWs; (iii) semi-permeable transparent dressings were used and changed when clinically indicated; (iv) PVCs were routinely replaced every four days.

**Supplementary results**

**e-Figure 1:** Inclusions and exclusions

**
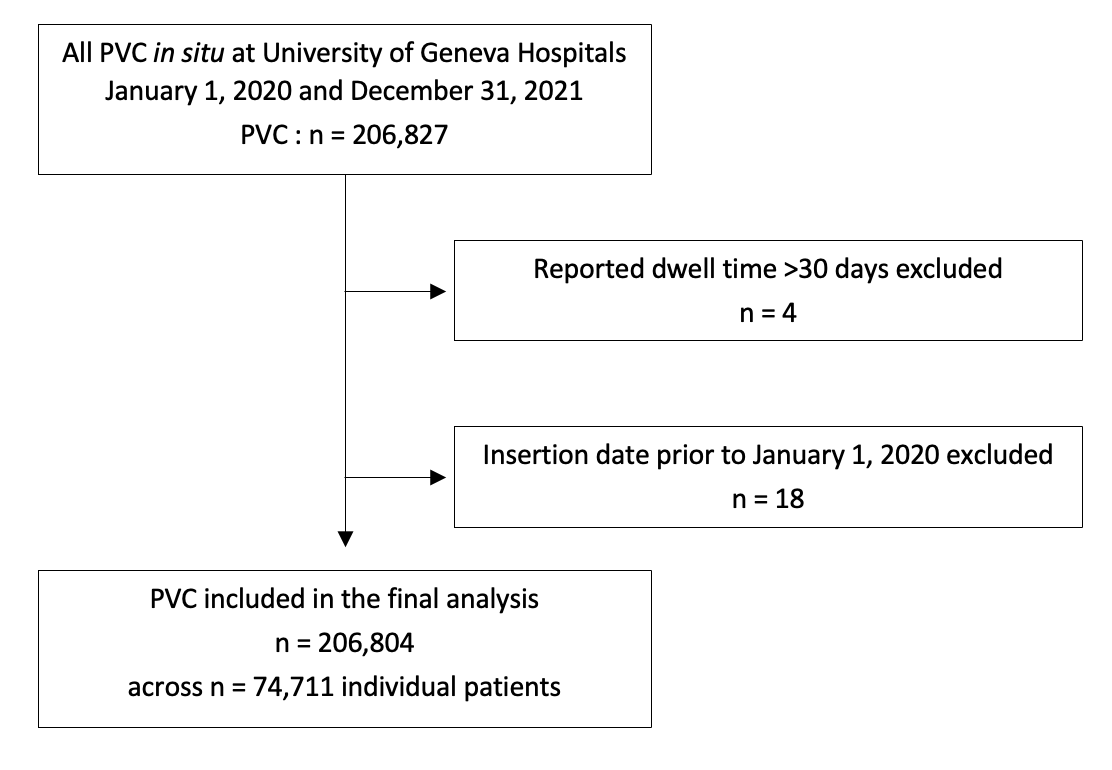
**

**e-Figure 2:** Hospitalisations with a diagnosis of COVID-19 at Geneva University Hospitals: January 1, 2020 – December 31, 2021


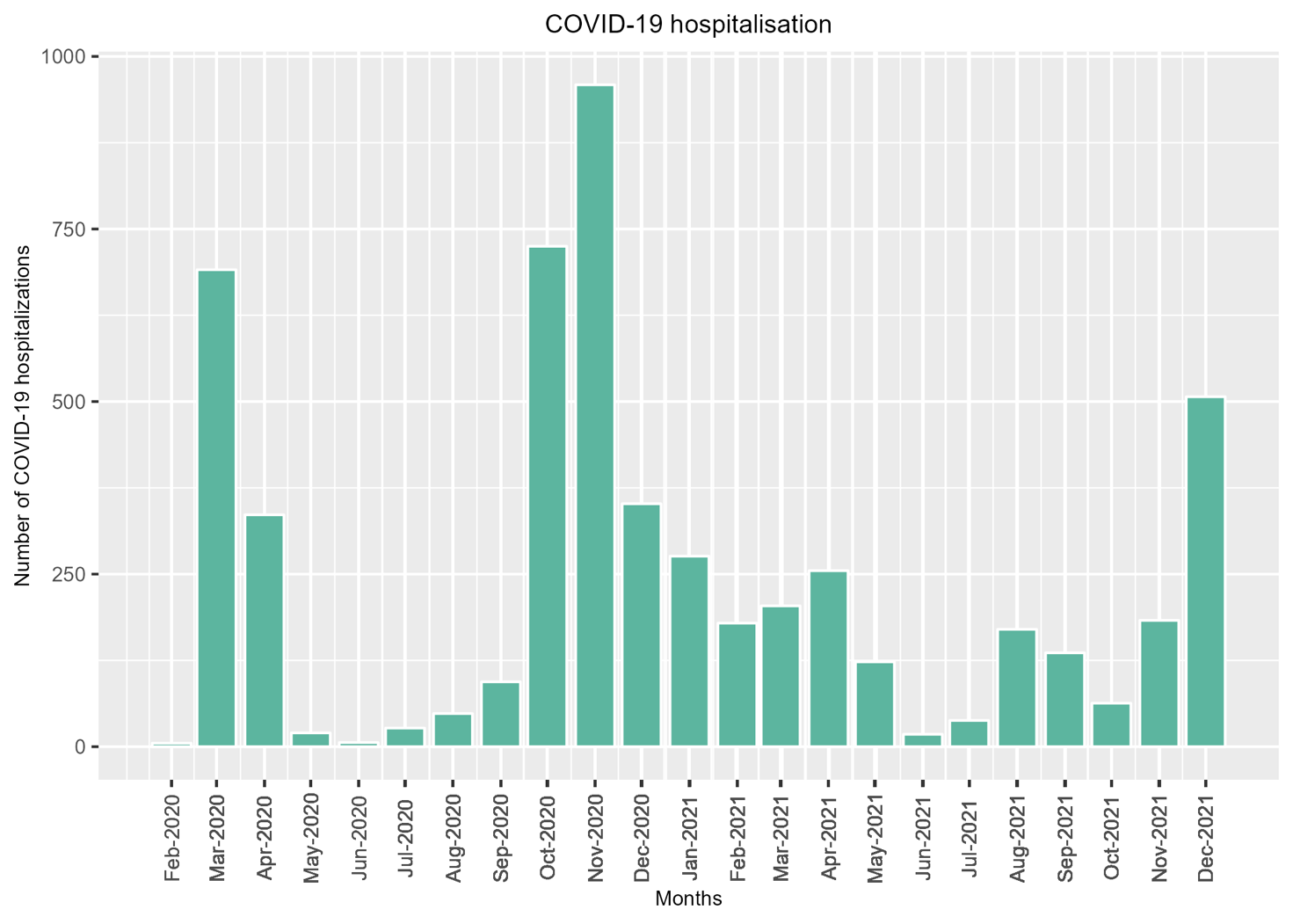


**e-Table 1: Characteristics of all PVC inserted in 2020 and 2021**

|  |  | 2020  (n=101,550) | | 2021  (n=105,254) | | p-value |
| --- | --- | --- | --- | --- | --- | --- |
| Sex | |  |  |  |  | <0.01 |
|  | Female | 50,569 | (49.8) | 54,018 | (51.3) |  |
|  | Male | 50,981 | (50.2) | 51,236 | (48.7) |  |
| Age, median (IQR) | | 66 | (46, 82) | 64 | (43, 81) | <0.01 |
| ICU admission | | 3,925 | (3.9) | 1,475 | (1.4) | <0.01 |
| Catheter size | |  |  |  |  | <0.01 |
|  | <= 16G | 5,997 | (6.1) | 4,809 | (4.6) |  |
|  | 18G | 38,714 | (39.7) | 41,560 | (40) |  |
|  | 20G | 44,358 | (45.4) | 48,689 | (46.9) |  |
|  | >= 22G | 8,558 | (8.8) | 8,728 | (8.4) |  |
| Time to catheter insertion, median (IQR) | | 0 | (0, 4) | 0 | (0, 3) | <0.01 |
| Dwell-time, median (IQR) | | 1.7 | (0.8, 3.5) | 1.7 | (0.65, 3.5) | <0.01 |
| Insertion site | |  |  |  |  | <0.01 |
|  | Arm | 1,865 | (1.8) | 1,802 | (1.7) |  |
|  | Cubital fossa | 19,830 | (19.5) | 21,929 | (20.8) |  |
|  | Forearm | 37,633 | (37.1) | 38,464 | (36.5) |  |
|  | Wrist | 17,256 | (17) | 17,581 | (16.7) |  |
|  | Hand | 22,671 | (22.3) | 23,417 | (22.2) |  |
|  | External jugular | 187 | (0.2) | 168 | (0.2) |  |
|  | Leg/foot | 1,922 | (1.9) | 1,755 | (1.7) |  |
|  | Other | 186 | (0.2) | 138 | (0.1) |  |
| PVC-BSI | | 10 | (0) | 27 | (0) | <0.01 |

PVC peripheral venous catheter G gauge IQR interquartile range ICU intensive care unit
PVC-BSI Peripheral venous catheter-associated bloodstream infection

**e- Table 2: Characteristics of PVC associated with PVC-BSI, inserted in 2020 and 2021**

|  |  | | 2020  (n=10) | | 2021  (n=27) | | p-value |
| --- | --- | --- | --- | --- | --- | --- | --- |
| Sex | | |  |  |  |  | 0.62 |
|  | Female | | 3 | (30) | 6 | (22) |  |
|  | Male | | 7 | (70) | 21 | (78) |  |
| Age, median (IQR) | | | 70.5 | (58, 86) | 67 | (51, 74) | 0.29 |
| Ward of insertion | | |  |  |  |  | 0.81 |
|  | Surgical | | 2 | (20) | 6 | (22) |  |
|  | Emergency department | | 8 | (80) | 20 | (74) |  |
|  | Other ward | | 0 | (0) | 1 | (4) |  |
| ICU admission (%) | | | 2 | (20) | 9 | (33) | 0.43 |
| BMI, median (IQR) | | | 24 | (21, 27) | 25 | (24, 28) | 0.67 |
| Charlson Score, median (IQR) | | | 5 | (1, 6) | 4 | (2, 8) | 0.60 |
| CABSI detection in a COVID-19 unit | | | 5 | (50) | 5 | (19) | 0.06 |
| Patient COVID-19 positive | | | 5 | (50) | 5 | (19) | 0.06 |
| Timing of catheter insertion from admission, median (IQR) | | | 6.5 | (2, 13) | 1 | (0, 4) | 0.09 |
| Catheter dwell-time, median (IQR) | | | 4.2 | (3.8, 6.1) | 4.2 | (2.1, 5.1) | 0.49 |
| Insertion site | | |  |  |  |  | 0.24 |
|  | Arm | | 0 | (0) | 1 | (4) |  |
|  | Cubital fossa | | 2 | (20) | 4 | (15) |  |
|  | Forearm | | 3 | (30) | 9 | (33) |  |
|  | Wrist | | 4 | (40) | 3 | (11) |  |
|  | Hand | | 1 | (10) | 10 | (37) |  |
| Microbiology | | |  |  |  |  | 0.29 |
|  | Gram positive | | 6 | (60) | 15 | (55) |  |
|  |  | Coagulase-negative staphylococci | 4 | (40) | 10 | (37) |  |
|  |  | *Staphylococcus aureus* | 1 | (10) | 5 | (19) |  |
|  |  | Other Gram-positive | 1 | (10) | 1 | (4) |  |
|  | Gram negative | | 2 | (20) | 7 | (26) |  |
|  |  | Enterobacterales | 2 | (20) | 6 | (22) |  |
|  |  | Other Gram-negative | 0 | (0) | 1 | (4) |  |
|  | Polymicrobial | | 0 | (0) | 4 | (15) |  |
|  | Fungi | | 2 | (20) | 1 | (4) |  |

PVC peripheral venous cannula, CABSI catheter-related bloodstream infection ICU intensive care unit
 IQR interquartile range

**e-Table 3: Univariable marginal Cox model for PVC-BSI among all PVC (n=101,550) in 2020**

|  |  | Without PVC-BSI (n = 101,540) | |  | PVC-BSI  (n = 10) | |  | HR | 95% CI | p-value |
| --- | --- | --- | --- | --- | --- | --- | --- | --- | --- | --- |
| Sex |  |  |  |  |  |  |  |  |  |  |
|  | Female (%) | 50,566 | (49.8) |  | 3 | (30) |  | 0.56 | (0.11, 2.96) | 0.50 |
|  | Male (%) | 50,974 | (50.2) |  | 7 | (70) |  |  |  |  |
| Age >66 years (%) | | 50,699 | (49.9) |  | 5 | (50) |  | 0.68 | (0.15, 3.06) | 0.61 |
| ICU admission (%) | | 3,923 | (3.9) |  | 2 | (20) |  | 6.71 | (1.29, 34.9) | 0.02 |
| Catheter Size* | |  |  |  |  |  |  |  |  |  |
|  | <=18G | 44,707 | (45.8) |  | 4 | (40) |  | 0.932 | (0.23, 3.83) | 0.92 |
|  | >=20G | 52,910 | (54.2) |  | 6 | (60) |  |  | |  |
| Time to insertion from admission, median (IQR) | | 1 | (1, 5) |  | 7.5 | (3, 14) |  | 0.997 | (0.99, 1.00) | 0.40 |
| Insertion site | |  |  |  |  |  |  |  |  |  |
|  | Cubital fossa | 19,828 | (19.5) |  | 2 | (20) |  | 0.296 | (0.1, 3.1) | 0.50 |
|  | Forearm | 37,630 | (37.1) |  | 3 | (30) |  | 0.354 | (0.08, 1.49) | 0.16 |
|  | Hand | 22,670 | (22.3) |  | 1 | (10) |  | 0.296 | (0.03, 2.92) | 0.30 |
|  | Other | 21,412 | (21.1) |  | 4 | (40) |  |  | |  |

*Missing data for catheter size in 3,923 episodes

**e-Table 4: Univariable marginal Cox model for PVC-BSI among all PVC (n=206,804) in 2020-2021**

|  |  | Without PVC-BSI  (n = 206,727) | |  | PVC-BSI  (n = 37) | |  | HR | 95% CI | p-value |
| --- | --- | --- | --- | --- | --- | --- | --- | --- | --- | --- |
| Year 2021 (%) | | 105,227 | (50.9) |  | 27 | (73) |  | 2.73 | (1.19, 6.29) | 0.02 |
| Sex |  |  |  |  |  |  |  |  |  |  |
|  | Female (%) | 104,578 | (50.6) |  | 9 | (24.3) |  | 0.39 | (0.17, 0.90) | 0.03 |
|  | Male (%) | 102,189 | (49.4) |  | 28 | (75.7) |  |  |  |  |
| Age >66 years (%) | | 99,634 | (48.2) |  | 20 | (54.1) |  | 0.91 | (0.45, 1.84) | 0.80 |
| ICU admission (%) | | 5,389 | (2.6) |  | 11 | (29.7) |  | 16.3 | (7.93, 33.63) | <.01 |
| Catheter Size* | |  |  |  |  |  |  |  |  |  |
|  | <=16G | 80262 | (39.9) |  | 12 | (34.3) |  | 1.56 | (0.35, 6.91) |  |
|  | 18G | 93031 | (46.2) |  | 16 | (45.7) |  | 1.54 | (0.35, 6.84) |  |
|  | 20G | 10801 | (5.4) |  | 5 | (14.3) |  | 4.52 | (1.14, 18.0) |  |
|  | >=22G | 17284 | (8.6) |  | 2 | (5.7) |  |  |  | 0.07 |
| Time to insertion from admission, median (IQR) | | 1 | (1, 4) |  | 3 | (1, 6) |  | 0.99 | (0.99, 1.00) | 0.29 |
| Insertion site | |  |  |  |  |  |  |  |  |  |
|  | Cubital fossa | 41,753 | (20.2) |  | 6 | (16.2) |  | 0.824 | [0.26-2.61] |  |
|  | Forearm | 3,666 | (1.8) |  | 1 | (2.7) |  | 1.092 | [0.13-9.22] |  |
|  | Hand | 46,077 | (22.3) |  | 11 | (29.7) |  | 1.576 | [0.57-4.39] |  |
|  | Other | 39,186 | (19.0) |  | 7 | (18.9) |  |  | | 0.42 |

*Missing data for catheter size in 5,391 episodes

**Supplementary discussion**

Increasing incidence of central line associated bloodstream infections (CLABSI) during COVID-19 pandemic have been reported in single-centre studies and multicentre surveillance data [5-9]. These include surveillance data from the United States reported to the Centers for Disease Control and Prevention/National Healthcare Safety Network [5, 7], and from the Dutch national surveillance system PREZIES [10]. In addition, a consortium of seven low and middle income countries reporting data to the International Nosocomial Infection Control Consortium (India, Mongolia, Jordan, Lebanon, Palestine, Egypt, and Turkey) reported increased CLABSI rates after the beginning of the COVID-19 pandemic. [6] Similar findings have been reported in single centre studies in the United States [8] and Europe [9]. These studies all reported a significant increase in CLABSI rates afer the beginning of the COVID-19 pandemic. However, they primarily examine data only from 2020, and have limited analysis of patient-level data.

One study of German surveillance data reported no increase in CLABSI during the COVID-19 pandemic. However, this analysis was limited to intensive care units, and analysed only data from 2020.[11]

**Supplementary references**

1. European Centre for Disease Prevention and Control. *Surveillance of healthcare-associated infections and prevention indicators in European intensive care units*. 2017 Available from: <https://ecdc.europa.eu/sites/portal/files/documents/HAI-Net-ICU-protocol-v2.2_0.pdf>.

2. Swiss Confederation Hospital-based COVID-19 Surveillance (CH-SUR). *Hospital-based surveillance of COVID-19 in Switzerland.* . 2022 31.12.2023 [cited 2023 14.05.2023]; Available from: <https://www.unige.ch/medecine/hospital-covid/>.

3. Thiabaud, A., et al., *Cohort profile: SARS-CoV-2/COVID-19 hospitalised patients in Switzerland.* Swiss medical weekly, 2021. **151**: p. w20475.

4. Buetti, N., et al., *Comparison of routine replacement with clinically indicated replacement of peripheral intravenous catheters.* JAMA Internal Medicine, 2021. **181**(11): p. 1471-1478.

5. Baker, M.A., et al., *The Impact of Coronavirus Disease 2019 (COVID-19) on Healthcare-Associated Infections.* Clinical Infectious Diseases, 2021. **74**(10): p. 1748-1754.

6. Rosenthal, V.D., et al., *The impact of COVID-19 on health care–associated infections in intensive care units in low- and middle-income countries: International Nosocomial Infection Control Consortium (INICC) findings.* International Journal of Infectious Diseases, 2022. **118**: p. 83-88.

7. Weiner-Lastinger, L.M., et al., *The impact of coronavirus disease 2019 (COVID-19) on healthcare-associated infections in 2020: A summary of data reported to the National Healthcare Safety Network.* Infection Control & Hospital Epidemiology, 2022. **43**(1): p. 12-25.

8. Ben-Aderet, M.A., et al., *Characterizing the relationship between coronavirus disease 2019 (COVID-19) and central-line–associated bloodstream infection (CLABSI) and assessing the impact of a nursing-focused CLABSI reduction intervention during the COVID-19 pandemic.* Infection Control & Hospital Epidemiology, 2022: p. 1-8.

9. Pérez-Granda, M., et al., *Increase in the frequency of catheter-related bloodstream infections during the COVID-19 pandemic: a plea for control.* Journal of Hospital Infection, 2022. **119**: p. 149-154.

10. Verberk, J.D., et al., *Healthcare-associated infections in Dutch hospitals during the COVID-19 pandemic.* Antimicrobial Resistance & Infection Control, 2023. **12**(1): p. 1-11.

11. Geffers, C., et al., *No increase of device associated infections in German intensive care units during the start of the COVID-19 pandemic in 2020.* Antimicrobial Resistance & Infection Control, 2022. **11**(1): p. 67.
